# State Lunatic Asylum, Austin

**Published:** 1899-03

**Authors:** 


STATE LUNATIC ASYLUM, AUSTIN.—We have received a handsomely illustrated copy of the "37th Annual Report of the Superintendent of the State Lunatic Asylum, at Austin, Texas" (Dr. B. M. Worsham, Superintendent), for the year ending October 31, 1898.

The institution seems to be pretty full. Its capacity is 700, and there were "remaining on hand October 31, 737," or 37 more patients than the asylum will hold. The daily average population was 722. There were discharged during the year: restored, 48; improved, 17; not improved, 7; total, 72. Admitted during the year, 119. There were applications made during the year for the admittance of 310 patients; 191 had to be refused on account of want of accommodations. The superintendent urges upon the Legislature the necessity of making an appropriation for additional buildings, and the board of managers strongly endorse the recommendation, and point out that the unfortunates who are not admitted to the asylums are "suffering torture in the common jails of the State." This is a shame, and a reproach which ought to be wiped out. There is a big surplus in the treasury; Texas has plenty of money.

The average cost of maintenance per capita was $140.83; the lowest this institution has ever had. The percentage of deaths to number treated was 3.68. Percentage of recoveries to whole number treated was 5.70. The products of the institution as per report of the supervisor, was, dairy, $3,539.04; farm, $6,105.50; mattresses, $778.25; garden, $1,954.88—total, $12,377.67. The total expenditures for the year were $120,068.40, of which $18,-388.50 was for repairs, stock on hand, etc. The administration is a very creditable showing for the superintendent, and is commended by the board of managers.

---

MENINGITIS is prevailing in several places in Texas, notably at Fort Worth, where it is reported to be very fatal; also at Dallas. We have not heard of any quarantines or shot-guns, notwithstanding the disease is contagious and very fatal. The memorable epidemic at Aurora, Texas, in January and February, 1890, (for an account of which see Dr. Burch's paper in the Transactions of the Texas State Medical Association, 1890, and also one by Dr. E. J. Beall, of Fort Worth, Texas, in TEXAS MEDICAL JOURNAL, October, 1891), was produced by the sheets and other articles from the bed of one Jefferson, who died in the Indian Territory, and by the contents of valise brought by the surviving Jeffersons to Aurora. Fifty per cent. of cases at Aurora died, and half of those who recovered were blind or lost their toes or both.

That the disease is produced by a *bacillus* and can be communicated as are other specific epidemic diseases, seems conclusive. It comes, therefore, under the head of "preventable diseases" and "quarantinable diseases," and ought to be prevented. The germ has never been discovered, and in Texas we are not aware that any effort is being made to find it. The appropriation this year for the quarantine department, we are informed, is $62,500, $15,000 of which is to build a floating quarantine warehouse and disinfecting plant at Galveston, as the costly warehouse built a few years ago on Pelican Spit is high and dry. These arrangements are to keep out yellow fever. No facilities for preventing meningitis, typhoid fever, consumption, diphtheria, etc., are provided by the State. Meantime, smallpox has gotten under headway at Laredo; trains are stopped, and Governor Sayers has asked for the loan of 200 United States tents, to be used by the State, and the State Health Officer will take charge of the Laredo epidemic.

